# Social capital and life satisfaction: a cross-sectional study on persons with musculoskeletal impairments in Hanoi, Vietnam

**DOI:** 10.1186/1471-2458-11-206

**Published:** 2011-04-01

**Authors:** Kyo Takahashi, Nguyen Thi Minh Thuy, Krishna C Poudel, Kayako Sakisaka, Masamine Jimba, Junko Yasuoka

**Affiliations:** 1 Department of Community and Global Health, Graduate School of Medicine, the University of Tokyo, 7-3-1 Hongo, Bunkyo-ku, Tokyo, Japan; 2 Department of Rehabilitation, Hanoi School of Public Health, 138 Giang Vo Str., Hanoi, Vietnam

## Abstract

**Background:**

Social capital has been recognized as a major social determinant of health, but less attention has been given to social capital of persons with musculoskeletal impairments. The present study aimed to explore the associations between social capital and life satisfaction of persons with musculoskeletal impairments in Hanoi, Vietnam.

**Methods:**

A cross-sectional study was conducted in Hanoi, Vietnam. From June to July 2008, we collected data from 136 persons with musculoskeletal impairments who belonged to disabled people's groups. Social capital was measured using a short version of the Adapted Social Capital Assessment Tool that included group membership, support from groups, support from individuals, citizenship activities, and cognitive social capital. Life satisfaction was measured using the Satisfaction with Life Scale. As possible confounding factors, we measured socio-economic factors and disability-related factors such as activities of daily living.

**Results:**

After controlling for confounding effects, group membership remained significantly associated with the level of life satisfaction reported by the persons with musculoskeletal impairments. In particular, being an active member of two or more groups was associated with higher life satisfaction. In contrast, other components of social capital such as citizenship activities and cognitive social capital were not significant in the multiple regression analysis of this study.

**Conclusions:**

The findings suggest the importance of considering an active participation in multiple groups toward the enhancement of the life satisfaction among persons with musculoskeletal impairments. To encourage persons with musculoskeletal impairments to have multiple active memberships, their access to groups should be facilitated and enhanced.

## Background

As the number of persons with disabilities increases, the social aspects of disability have gained substantial attention [1]. Disability is not just a negative consequence of a medical condition any longer but a complex situation with medical and social factors [2]. For example, 'full and effective participation and inclusion in society' is one of the main principles of the Convention on the Rights of Persons with Disabilities [3]. The state of persons with disabilities, however, varies by country [4]. The World Health Organization [5] has estimated that approximately 80 percent of persons with disabilities live in developing countries where the social aspects of disability still do not receive sufficient attention.

Social capital has been recognized as a major social determinant of health [6,7]. While the definition of social capital varies, Robert Putnam's definition [8] has been well accepted: 'features of social organization, such as trust, norms, and networks that can improve the efficiency of society by facilitating coordinating actions' [9]. However, social capital has not been investigated only as a social cohesion but with certain attributes of individual such as social support and social network [7]. For example, social capital has been divided into different types, with a common distinction being between structural and cognitive social capital [10]. Structural social capital, such as networks, is relatively tangible. On the other hand, cognitive social capital is invisible since it is measured by individuals' perceptions of connectedness in their community. The characteristics of these two types of social capital are dissimilar, but they robustly interact with each other [11]. In the health field, social capital has been studied mainly in the general population. For example, analyzing the data of 167,259 individuals in the United States, Kawachi et al. [12] revealed that low social capital was associated with self-rated poor health. Similarly, Helliwell and Putnam [13] confirmed that a strong link exists between social capital and subjective well-being in North America. Even in an ecological study, Bjornskov [14] found social capital to be a powerful factor in explaining the differences in levels of happiness among European countries. Despite the rapid accumulation of social capital studies, less attention has been paid to persons with disabilities who tend to be socially isolated.

Life satisfaction is one aspect of subjective well-being, and it can be a useful measure in health studies especially for persons with physical disabilities. Pavot and Diener [15] describe life satisfaction as 'a conscious cognitive judgment of one's life in which the criteria for judgment are up to the person.' In this sense, life satisfaction does not differentiate persons by physical ability or disability. In other words, life satisfaction can be properly measured regardless of whether persons are physically disabled. Despite the ample availability of instruments, most studies of the life satisfaction have been conducted in developed countries [16,17]. The various populations were targeted in those studies; however, the studies of the life satisfaction of persons with disabilities have been limited only to persons with spinal cord injuries [18-20].

Vietnam is a developing country experiencing rapid economic growth. It has sought to improve the social lives of persons with disabilities by promoting their participation in social activities. For example, the Hanoi Disabled People Association (DP Hanoi) was established in 2006 as the first legal organization of persons with disabilities. DP Hanoi consists of 22 various disabled people's groups whose members are persons with visual, hearing, or mobility disabilities. It has enlightened the human rights of persons with disabilities in Vietnam by putting up a number of events and has consistently encouraged persons with disabilities to actively participate in society by providing various opportunities including the useful information of employment. Although the social aspect of disability receives increasing attention elsewhere [21], little evidence regarding it is available from Vietnam and other developing countries.

Therefore, this study aimed to explore the association between social capital and life satisfaction of persons with physical disabilities in Hanoi, Vietnam.

## Methods

### Participants

In Vietnam, the number of persons with disabilities was estimated approximate 5,333,000 which was equivalent to 6.4% of the total population [4]. We targeted persons with disabilities who belonged to disabled people's groups in Hanoi, the capital city of Vietnam. The Ordinance on Disabled Persons in Vietnam defines persons with disabilities as those having 'defective of one or many parts of the body or functions which are shown in different forms of disability, and which reduce the capability of activity and cause many difficulties to work, life and studies [4].' The distribution of general types of disability in Vietnam was reported as follows: mobility 42%, learning 23%, hearing 22% and visual 7% in 2006 [22]. We particularly focused on musculoskeletal impairment, which could cause mobility disability and physically limit social participation.

Inclusion criteria included having one or more musculoskeletal impairments, belonging to a disabled people's group under DP Hanoi, and being over 18 years old. We excluded those with other kinds of disabilities such as visual, hearing, and intellectual disabilities.

Among 22 disabled people's groups under DP Hanoi, nine groups were run by and for persons with musculoskeletal impairments. Two hundred and thirty-two members were registered in the nine groups. We tried to contact all of them in the regular or special meetings of each group from June to July 2008. After the meetings, the group leaders contacted those who did not attend the meetings and recruited them individually. In the end, we contacted 152 members (65.5%) and 136 of them (58.6%) agreed to participate in this study and met the criteria.

We obtained ethical approval from the ethical committee of the University of Tokyo. We explained the study objectives to all participants, and obtained their informed consent. Two authors (KT and NTMT) supervised all aspects of the data collection.

### Instruments

We developed a self-administered questionnaire and had it translated from English into Vietnamese. The questionnaire was developed to measure social capital, life satisfaction, socio-economic factors, and disability-related factors. In February 2008, we pre-tested the questionnaire among 21 persons with musculoskeletal impairments in targeted disabled people's groups to enhance its validity and reliability. For those who could not write, we allowed them to be physically supported by personal assistants with their verbal responses.

#### Social capital

We used a short version of the Adapted Social Capital Assessment Tool (SASCAT) to measure the individual structural and cognitive social capital of the participants (Table 1). SASCAT was developed for use in studies in developing countries, including Vietnam [23], as a modified version of the Adapted Social Capital Assessment Tool [24]. Structural social capital was measured with five questions about 'group membership,' 'support from groups,' 'support from individuals,' 'joining together with other community members,' and 'talking with a local authority or government organization.' In the question regarding 'group membership,' we measured self-defined active participation. Those who did not actively participate in their group were not classified as active members regardless of their membership. Cognitive social capital was measured with four questions about 'trust,' 'social harmony,' 'sense of belonging,' and 'sense of fairness.' We defined 'community' as the official commune which was clearly understood as a geographical community in Vietnam [9,25].

**Table 1:** Short version of Adapted Social Capital Assessment Tool (SASCAT)

Questions		Coding
**Group membership**		0
1. In the last 12 months have you been an active member of any of the following types of groups in your community?		1
		2 or more
➣ Work related/trade union	➣ Credit/funeral group	
➣ Community association/co-op	➣ Sports group	
➣ Political group	➣ Others: specify	
➣ Religious group		
**Support from groups**		0
2. In the last 12 months, did you receive from the group any emotional help, economic help or assistance in helping you know or do things?		1
		2 or more
➣ Work related/trade union	➣ Credit/funeral group	
➣ Community association/co-op	➣ Sports group	
➣ Political group	➣ Others: specify	
➣ Religious group		
**Support from individuals**		0
3. In the last 12 months, have you received any help or support from any of the following, this can be emotional help, economic help or assistance in helping you know or do things?		1
		2 or more
➣ Family	➣ Friends who are not neighbours	
➣ Neighbours	➣ Government officials/civil service	
➣ Community leaders	➣ Charitable organizations/NGO	
➣ Religious leaders	➣ Other: specify	
➣ Politicians		
**Citizenship activities**		None
4. In the last 12 months, have you joined together with other community members to address a problem or common issues?		Joined or talked
5. In the last 12 months, have you talked with a local authority or governmental organization about problems in this community?		Joined & talked
Cognitive social capital*		
6. In general, can the majority of people in this community be trusted?		Yes = 1, No = 0
7. Do the majority of people in this community generally get along with each other?		Yes = 1, No = 0
8. Do you feel as though you are really a part of this community?		Yes = 1, No = 0
9. Do you think that the majority of people in this community would try to take advantage of you if they got the chance?		Yes = 0, No = 1

* Total score of four questions of cognitive social capital: 2 or less = low, 3 or 4 = high

#### Life satisfaction

To measure life satisfaction, we used the Satisfaction with Life Scale (SWLS), which was developed by Diener et al. [26]. A series of validation studies demonstrated satisfactory content and predictive validity among various age groups [26,27]. SWLS has been used for a wide variety of study purposes [28]. SWLS consists of five Likert items with seven response levels ranging from 'strongly disagree (= 1)' to 'strongly agree (= 7).' The items were: 1) In most ways my life is close to ideal, 2) The conditions of my life are excellent, 3) I am satisfied with my life, 4) So far I have gotten the important things I want in life, and 5) If I could live my life over, I would change almost nothing. The total score of SWLS ranged from 5 to 35, which we treated as a continuous variable. A high score was interpreted as high life satisfaction. Good internal consistency was observed with a Cronbach alpha coefficient of .87, which was close to .85 reported by the scale developers [26].

#### Socio-economic and disability-related factors

We measured socio-economic factors and disability-related factors as possible confounders. Socio-economic factors included age, sex, number of family members living with the participant, marital status, educational level, job status, individual income status, and war experience. Disability-related factors in this study were number of years with impairment, timing of commencement of impairment, number of impaired body part(s), and level of independence with respect to activities of daily living (ADL), indoor moving status, and outdoor moving status. We divided impaired body parts into six: neck, trunk, upper right limb, upper left limb, lower right limb, and lower left limb. Regarding ADL, we used the Barthel Index [29] which consists of 10 ADL-related items such as level of independence in toilet use, bathing, and dressing. We coded those who had a maximum score on the Barthel Index as 'independent' and the others as 'with help,' respectively. With regard to indoor moving status and outdoor moving status, we coded those who never used any assistive device or someone's help as 'independent.' On the other hand, those who used either or both of them were coded as 'with help.'

### Statistical analysis

We conducted two kinds of statistical analysis. First, we performed bivariate tests including independent-samples t-test and one-way analysis of variance (ANOVA) to explore the crude association between life satisfaction and all variables. Then, we ran a standard multiple regression to control for confounding effects. In the regression model, we included all social capital variables and the variables with *p*-values of less than .10 from the bivariate analysis. We treated the social capital variables except for 'citizenship activities' and ADL as continuous variables unlike in bivariate tests. SPSS version 16.0 for Windows (SPSS Inc., Chicago, IL) was used for all statistical analyses.

## Results

### Characteristics of the participants

Table 2 shows the characteristics of the 136 participants by gender. The mean age was 36 (SD 13.3) years old. All study participants were literate and 31 (22.8%) had graduated from a university. One hundred and seven (78.7%) had a job such as bicycle repair and shop assistant, and 80 (58.8%) had a regular job or lived on a pension, which provides regular income. The causes of participants' impairments varied such as polio, angiopathy, and injury by traffic accidents or war; accordingly, types of impairment varied such as deformation, amputation, and paralysis. Of the total, 43 (31.6%) were disabled at birth, and 70 (51.5%) had two or more disabled parts. Ninety (66.2%) who had the maximum score of the Barthel Index did not need any help with ADL.

**Table 2:** Characteristics of the participants (*n *= 136)*

Variable		Male (*n *= 68)	Female (*n *= 68)
		*n *(%)	*n *(%)
**Socio-economic variable**			
Age	Mean (SD)	38.3 (14.7)	33.5 (11.4)
*n *of family members	Mean (SD)	4.5 ( 2.3)	4.0 ( 2.1)
Marital status	Single	31 (45.6)	49 (72.1)
	Married	30 (44.1)	9 (13.2)
	Others^†^	5 ( 7.4)	6 ( 8.8)
Educational level	< High school	27 (39.7)	25 (36.8)
	High school	21 (30.9)	22 (32.4)
	University	16 (23.5)	15 (22.1)
Job	Yes	52 (76.5)	55 (80.9)
	No	16 (23.5)	13 (19.1)
Regular income	Yes	40 (58.8)	40 (58.8)
	No	27 (39.7)	25 (36.8)
War experience	Yes	8 (11.8)	1 ( 1.5)
	No	58 (85.3)	66 (97.1)
**Disability-related variable**			
Impaired years	Mean (SD)	24.4 (14.7)	25.8 (13.1)
Timing of impairment	At birth	24 (35.3)	19 (27.9)
	Later	43 (63.2)	46 (67.6)
*n *of impaired part^‡^	1	32 (47.1)	34 (50.0)
	2 or more	36 (52.9)	34 (50.0)
Activities of daily living	Independent	47 (69.1)	43 (63.2)
	With help	15 (22.1)	12 (17.6)
Indoor moving status	Independent	42 (61.8)	45 (66.2)
	With help	25 (36.8)	21 (30.9)
Outdoor moving status	Independent	28 (41.2)	32 (47.1)
	With help	37 (54.4)	36 (52.9)

* Missing cases are not shown in this table.

^† ^Widowed or divorced

^‡ ^Impaired parts were divided into six: neck, trunk, upper right limb, upper left limb, lower right limb, and lower left limb.

### Life satisfaction of persons with musculoskeletal impairments

In the binary analysis, we found significant associations between several variables and life satisfaction. As shown in Table 3, educational level (*p *= .009) was significantly associated with life satisfaction. Those with high education, especially the university graduates, were more likely to have higher life satisfaction. Regarding disability-related factors, ADL (*p *= .013) and indoor moving status (*p *= .015) were significantly associated with life satisfaction. Those who were physically independent were inclined to have higher life satisfaction.

**Table 3:** Association between socio-economic and disability-related variables and life satisfaction

Variable		Mean*	SD	***p***^†^
Age	<30	17.5	6.3	.208
	30 - 39	15.3	6.7	
	40 - 49	15.4	4.5	
	>49	18.1	5.0	
Sex	Male	16.8	6.0	.744
	Female	17.2	5.9	
Educational level	<High school	15.3	4.9	**.009**
	High school	16.7	4.9	
	University	19.4	7.8	
Job	Yes	17.2	5.9	.484
	No	16.3	6.0	
Regular income	Yes	17.5	6.0	.177
	No	16.0	5.8	
Activities of daily living	Independent	17.7	5.9	**.013**
	With help	14.4	5.7	
Indoor moving status	Independent	18.0	6.0	**.015**
	With help	15.3	5.5	
Outdoor moving status	Independent	18.1	5.7	.086
	With help	16.3	6.1	

* Mean score of the Satisfaction with Life Scale (SWLS)

^† ^One-way analysis of variance (ANOVA) for age and educational level; independent-samples t-test for other variable

Table 4 demonstrates the association between social capital and life satisfaction. Out of the five items of structural social capital, 'group membership' (*p *< .001) and 'support from groups' (*p *= .048) were significantly associated with life satisfaction. Those who were active members of two or more groups had higher life satisfaction than those who actively participated in only one group or who did not actively participate in any groups. Similarly, those who received support from two or more groups were inclined to have higher life satisfaction. Regarding the four items of cognitive social capital, 'sense of belonging' (*p *= .017) to their community was observed as a significant contributor to high life satisfaction. The one hundred and seven participants (78.7%) who felt themselves to be a part of their community were more likely to have higher life satisfaction.

**Table 4:** Association between social capital components and life satisfaction

Variable		*n *(%)	Mean *	SD	***p***^**†**^
Group membership	0	44 (32.4)	16.0	3.7	**<.001**
	1	58 (42.6)	16.0	6.2	
	2 or more	27 (19.9)	20.9	6.7	
Support from groups	0	57 (41.9)	16.4	4.8	**.048**
	1	51 (37.5)	16.5	5.8	
	2 or more	21 (15.4)	19.9	7.8	
Supports from individuals	0	27 (19.9)	15.4	3.4	.183
	1	35 (25.7)	16.6	5.3	
	2 or more	67 (49.3)	17.9	6.8	
Citizenship activities	None	48 (39.7)	16.6	6.5	.628
	Joined or talked	17 (14.0)	17.2	7.3	
	Joined & talked	56 (46.3)	17.8	4.9	
Joined with others	Yes	64 (47.1)	17.8	4.9	.335
	No	59 (43.4)	16.7	6.7	
Talked with authorities	Yes	67 (49.3)	17.6	5.5	.428
	No	55 (40.4)	16.8	6.4	
Cognitive social capital	Low	22 (18.5)	15.6	4.5	.133
	High	97 (81.5)	17.7	6.2	
Trust	Yes	99 (72.8)	17.5	6.2	.217
	No	24 (17.6)	16.3	3.9	
Social harmony	Yes	97 (71.3)	17.4	6.1	.681
	No	26 (19.1)	16.9	4.9	
Sense of belonging	Yes	107 (78.7)	17.7	5.9	**.017**
	No	14 (10.3)	13.6	5.2	
Sense of fairness^‡^	Yes	19 (14.0)	16.5	4.5	.531
	No	103 (75.7)	17.4	6.1	

* Mean score of the Satisfaction with Life Scale (SWLS)

^† ^One-way analysis of variance (ANOVA) for group membership, support from groups, support from individuals, and citizenship activities; independent-samples t-test for other variables

^‡ ^This variable was reverse coded so 'No' indicates more social capital.

Table 5 shows the result of the multiple regression analysis. Since multicolinearity was found between 'group membership' and 'support from groups' (Pearson's correlation = .728), we excluded 'support from groups' from the regression model. The model's R^2 ^was .233, and the significance of the model was confirmed with ANOVA (*p *= .012). As a result, only 'group membership' was significantly associated with life satisfaction (Standardized Beta = .26, t = 2.01, *p *= .041). The effect of other variables was considerably weakened although 'educational level (university)' remained close to the significant level (Standardized Beta = .24, t = 1.82, *p *= .073).

**Table 5:** Multiple linear regression predicting life satisfaction by social capital components and significant variables* (*n *= 92)

Variable	**Multiple regression (R**^**2 **^**= 0.233)**				
	
	Unstandardized Coefficient		Standardized Coefficient		
			
	B	SE	Beta	t	*p*
**Social capital**^†^					
Group membership^‡^	1.73	0.83	.26	2.01	**.041**
Support from individuals^‡^	-0.08	0.53	-.02	-0.15	.879
Citizenship activities					
None	Reference				
Joined or talked	-1.10	1.94	-.06	-0.57	.573
Joined and talked	1.57	1.47	.12	1.07	.289
Cognitive social capital^‡^	0.64	0.67	.10	0.95	.347
**Socio-economic factor**					
Educational level					
< High school	Reference				
High school	1.04	1.64	.08	0.64	.527
University	3.22	1.78	.24	1.82	.073
**Disability-related factors**					
Activities of daily living^‡^	0.56	0.54	.12	1.03	.304
Indoor moving status					
With help	Reference				
Independent	2.35	2.01	.17	1.17	.246
Outdoor moving status					
With help	Reference				
Independent	-0.93	1.63	-.07	-0.57	.570

* Variables with *p*-values less than .10 in binary analysis

^† ^'Support from groups' was excluded from the regression model due to multicolinearity with 'group membership.'

^‡ ^Continuous variables

## Discussion

### Structural social capital and life satisfaction of persons with musculoskeletal impairments

In this study, one of the structural social capital components, 'group membership,' was significantly associated with life satisfaction even after controlling for confounding factors. Specifically, being an active member of two or more groups was associated with higher life satisfaction. This finding indicates that the number of groups to which an individual actively belongs could differentiate the life satisfaction. Those who had higher life satisfaction were active members of not only disabled people's groups but also other types of groups, such as religious groups and sports groups. Being an active member of these groups in addition to disabled people's groups could lead to higher life satisfaction. In contrast, those who thought they were not active members of any group and those who thought they were active members of only one group were more likely to have lower life satisfaction. Furthermore, this finding can be interpreted as showing a positive effect of social participation on life satisfaction. Persons with musculoskeletal impairments are more likely to be socially isolated because of their impairments and a potentially unfriendly environment around them, such as physical barriers to access or personal prejudice [30]. Whether persons with musculoskeletal impairments can participate in society through group activities, therefore, can significantly affect their life satisfaction.

Other components of structural social capital such as 'support from individuals' and 'citizenship activities' were not significantly associated with life satisfaction. Although direct evidence is lacking, we interpret these results as follows. Regarding 'support from individuals,' the term 'support' might have been understood narrowly by persons with musculoskeletal impairments. Our participants received physical support from others to the extent that they needed it. That situation might have caused them to think that 'support' meant just physical support. As for 'citizenship activities,' the inaccessibility to citizenship activities in Vietnam should be reconsidered [31]. In Vietnam, it is essential for citizens to have a connection with authorities such as head members of the Communist Party to make any action in their community. The participants who did not have any personal connection with authorities could have an opportunity to meet with authorities in certain official meetings which attached great importance to formality. However, they might know that the discussion in those meetings meant a little compared to personal connection with authorities. Similarly, distinct administrative division of community builds a barrier between communities. De Silva et al. [32] have pointed out the low level of 'citizenship activities' in Vietnam, especially related to talking to the authorities. Persons with musculoskeletal impairments could unilaterally make a decision related to 'group membership;' however, participation in 'citizenship activities' requires the willingness of others to collaborate. This difference may explain why 'citizenship activities' did not affect life satisfaction.

### Cognitive social capital and life satisfaction of persons with musculoskeletal impairments

Cognitive social capital plays a crucial role in mental health [33]; however, none of the cognitive social capital components were significant in our multiple regression analysis. Since cognitive social capital is related to one's subjective perception of the community, the term 'community' should be carefully defined. While we defined the term 'community' as the official commune in Vietnam, the perception of the official commune might be different from the 'community' especially in persons with musculoskeletal impairments. For example, we measured a sense of belonging to the community with the question 'Do you feel as though you are really a part of this community?'. However, persons with musculoskeletal impairments could feel a sense of belonging not only to the official commune but also to various types of informal communities. For instance, the virtual community of the Internet might be important because of its greater ease of access for some persons with musculoskeletal impairments [34]. To assess the actual impact of cognitive social capital on the life satisfaction of persons with musculoskeletal impairments, the appropriate definition of the term 'community' should be carefully considered.

### Educational level and life satisfaction of persons with musculoskeletal impairments

Persons with musculoskeletal impairments may face difficulties in continuing their education due to inaccessibility and discrimination [30]. The literacy rate has reached over 90% in Vietnam [22,35]; however, it is still not known how many persons with or without musculoskeletal impairments do not go to university and for what reasons. In Vietnam, universities provide useful opportunities for social participation. For example, two out of nine disabled people's groups that we targeted in this study were university-based, and the majority of members of those two groups were talked into joining the groups by peers in the same university. Although we did not find a significant association between educational level and life satisfaction in our multiple regression analysis probably because people with high education were likely to have more opportunities to participate in various groups such as sports group in university, equal access to higher education for persons with musculoskeletal impairments remains an important factor in their social participation.

### Limitations

The findings of this study, however, should be interpreted carefully. First, this study did not consider whether a participant could recover from the musculoskeletal impairment. Most participants were living with musculoskeletal impairments that were medically stable but likely permanent. Social factors may play a different role among persons with recoverable musculoskeletal impairments because they tend to focus more on medical factors. Second, this study was conducted only with the members of disabled people's groups in the capital city. Therefore, caution is needed to generalize our findings to the other populations such as persons without musculoskeletal impairments, persons with musculoskeletal impairments who do not belong to any group, and persons with musculoskeletal impairments in rural area. Third, our analysis does not show the relationship between specific combinations of groups and life satisfaction since it included data on the actual number of groups each participant actively participate in without making any combinations of groups. Fourth, this study was a cross-sectional study which cannot clarify the actual causality. Although we found a significant association between particular variables and life satisfaction, we must use additional research methods such as case studies and in-depth interviews, and further longitudinal studies are necessary to clarify the impact of social capital on life satisfaction.

## Conclusions

Despite these limitations, the study findings are relevant because we revealed the importance of social aspects of disability with quantitative data. In addition, this was the first social capital study to target persons with musculoskeletal impairments in Vietnam. We expect our findings could be leverage to improve the social situation of persons with musculoskeletal impairments especially in developing countries. The findings suggest the importance of considering an active membership of two or more groups to increase the life satisfaction of persons with musculoskeletal impairments. To encourage persons with musculoskeletal impairments to have multiple active memberships, their access to groups should be encouraged and enhanced. For example, information about community groups should be available in various formats such as via the Internet, and the meeting places should be easily accessible to persons with any type of musculoskeletal impairment. Furthermore, group members should make reasonable accommodation to welcome persons with musculoskeletal impairments in their group. Without adequate accessibility, persons with musculoskeletal impairments cannot participate in group activities no matter how highly they are motivated.

## Competing interests

The authors declare that they have no competing interests.

## Authors' contributions

KT conceived the research questions, designed the study, conducted preparatory field works, collected data, analyzed data, and drafted the manuscript for publication. NTMT was involved in revisions of the research proposal, preparatory field works, and data collection. KCP was involved in revisions of the research proposal, data analysis, and revisions of the manuscript for publication. KS was involved in revisions of the research proposal, data analysis, and revisions of the manuscript for publication. MJ was involved in revisions of the research proposal, data analysis, and revisions of the manuscript for publication. JY was involved in revisions of the research proposal, data analysis, and revisions of the manuscript for publication. All authors read and approved the final manuscript.

## Pre-publication history

The pre-publication history for this paper can be accessed here:

http://www.biomedcentral.com/1471-2458/11/206/prepub
